# Lethal Case of Bourbon Virus Leading to Shock and ECMO Utilization

**DOI:** 10.1155/crdi/4652171

**Published:** 2026-01-16

**Authors:** Allianna Mitchell, Andrew Gessouroun, Wissam El Atrouni, Ryan Kubat, Omar Almoghrabi, Megan Vorhies, Brigid Flynn

**Affiliations:** ^1^ School of Medicine, University of Kansas Medical Center, 3901 Rainbow Blvd, Kansas City, 66160, Kansas, USA, kumc.edu; ^2^ Department of Anesthesiology, University of Kansas Medical Center, 3901 Rainbow Blvd, Kansas City, 66160, Kansas, USA, kumc.edu; ^3^ Department of Internal Medicine, Division of Infectious Disease, University of Kansas Medical Center, 3901 Rainbow Blvd, Kansas City, 66160, Kansas, USA, kumc.edu; ^4^ Department of Cardiothoracic Surgery, University of Kansas Medical Center, 3901 Rainbow Blvd, Kansas City, 66160, Kansas, USA, kumc.edu; ^5^ Department of Emergency Medicine, Virginia Commonwealth University Health System, 1250 E. Marshall Street, Richmond, 23298, Virginia, USA, vcuhealth.org

**Keywords:** Bourbon virus, ECMO, *Mucor* pneumonia, shock, tick

## Abstract

We present a lethal case of Bourbon virus infection in a 63‐year‐old Caucasian, diabetic male who was previously in good health. The patient had spent time in the wooded areas of Bourbon County, Kansas, and removed three ticks from his body 5 days prior to presentation. The patient had acute multisystem organ failure requiring multiple inotropes and pressor agents, renal replacement therapy, and venoarterial extracorporeal membrane oxygenation (VA‐ECMO). This report describes the presentation, clinical outcomes, and background on Bourbon virus infection.

## 1. Introduction

Bourbon virus was first discovered in 2014 in a patient from Bourbon County, Kansas, USA, in an index patient who also expired from his illness at our institution [1]. Since its discovery, cases of Bourbon virus have been reported in only four states: Kansas, Oklahoma, Missouri, and New York [2]. While human infections are rarely reported, numerous other states have confirmed the detection of the Bourbon virus in nonhuman mammal hosts [1]. Bourbon virus has been detected in all life stages (larvae, nymph, and adult) of lone star ticks (*Amblyomma americanum*) [3] (Figure 1). The current case report is the sixth report of Bourbon virus infecting humans.

Figure 1The female lone star tick, *Amblyomma americanum*, is seen in image (a). (b) Image depicts a lone star tick, *Amblyomma americanum* nymph, that was crawling on a person’s fingernail [4, 5]. Figure courtesy of the Public Health Image Library (https://phil.cdc.gov).

**Figure:**
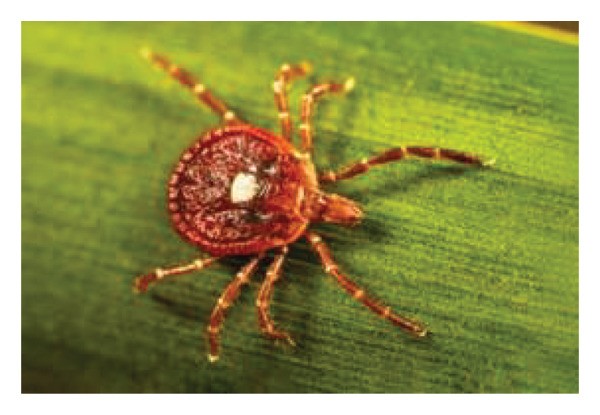
(a)

**Figure:**
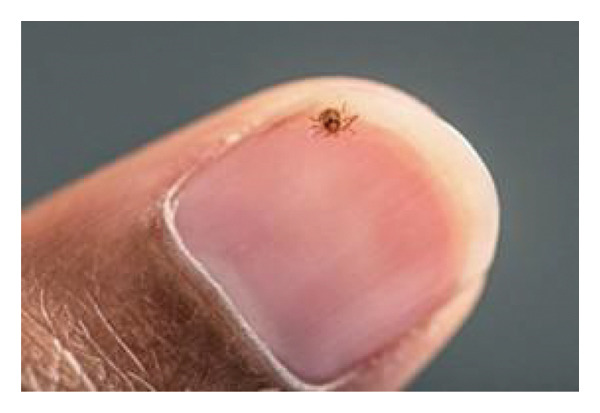
(b)

## 2. Case Presentation

A 63‐year‐old man with a past medical history of well‐controlled insulin‐dependent diabetes (hemoglobin A1c of 5.9%), hypertension, hyperlipidemia, and hypothyroidism presented to an urgent care clinic due to near syncope while standing, along with two days of mild headache, myalgias, poor appetite, and lower extremity edema. Seven days before the presentation, he was preparing hunting grounds during springtime in Bourbon County, Kansas, USA. He had removed two small, black, nonengorged ticks from his body that day and another small, black, nonengorged tick two days later from his lower back, with the assumption that this last tick was attached for approximately 72 h. Upon returning home from the wooded area, he began having neck pain.

At the urgent care clinic, he was hypotensive with a blood pressure of 67/54 mmHg and was transferred to our tertiary care hospital and admitted to the intensive care unit (ICU). Upon arrival at the ICU, he complained of severe neck pain and dizziness. He had hypothermia of 34.6°C that failed to correct despite warming attempts, hypotension, lactic acidosis, acute kidney injury, and multiple organ failure. Initial laboratory testing demonstrated hemoglobin of 17.9 g/dL (13.5–16.5 g/dL), platelets of 113,000 cells/μL (150,000–400,000 cells/μL), white blood cells of 31,200 cells/μL (4.5–11.0 cells/μL) with 88% or 27.3 k/μL neutrophils (41%–77%) and 6% or 1.93 k/μL lymphocytes (24%–44%), sodium of 127 mmol/L (137–147 mmol/L), potassium of 6.1 mmol/L (3.5–5.1 mmol/L), carbon dioxide (CO_2_) of 14 mmol/L (21–30 mmol/L), creatinine of 4.6 mg/dL (0.4–1.24 mg/dL), creatinine kinase of 498 U/L (35–232 U/L), aspartate aminotransferase (AST) of 46 U/L (7–40 U/L), alanine aminotransferase (ALT) of 17 U/L (7–56 U/L), and lactic acid of 4.5 mmol/L (0.5–2.0 mmol/L). Initial transthoracic echocardiogram demonstrated left ventricular ejection fraction of 55%.

Aggressive volume resuscitation, broad‐spectrum antibiotics, including doxycycline for potential bacterial tick‐borne illness, and high doses of vasopressors and inotropes were started. Despite volume resuscitation, his acidosis required initiation of continuous renal replacement therapy. He sustained several hypotensive episodes, one where his blood pressure was 38/20. Due to the lack of a pulmonary artery catheter, we were unable to ascertain cardiac indices or other variables. Within 4 hours of ICU admission, he was on maximum doses of norepinephrine (0.5 mcg/kg/min), phenylephrine (3 mcg/kg/min), vasopressin (2.4 U/hr), and epinephrine (1 mcg/kg/min), which had been quickly up‐titrated in order to achieve hemodynamic goals. He became unresponsive and was endotracheally intubated for airway protection and worsening shock. Serial transthoracic echocardiograms demonstrated an increasingly worse left ventricular ejection fraction from 45% to 5% within a few hours. His lactic acid peaked at 20 mmol/L.

He was emergently cannulated for peripheral venoarterial extracorporeal membrane oxygenation (VA‐ECMO) and Impella CP (Abiomed, Danvers, MA) for left ventricular unloading, in hopes of end‐organ stabilization and recovery. At this time, his laboratory evaluations were notable for white blood cells of 31,800 cells/μL (eventually peaking at 52,000 cells/μL), platelets of 68,000 cells/μL, AST of 1060 U/L, ALT of 512 U/L, and creatinine kinase of 44,300 U/L. Endomyocardial biopsy was performed on Hospital day 2. Results were promptly reported; however, they were negative. Coronary catheterization demonstrated only mild, nonobstructive coronary artery disease.

Initial extensive infectious workup was negative for all preliminary tested specimens, including blood and respiratory cultures and viral cultures (*Ehrlichia*, *Anaplasma*, Epstein–Barr virus, and cytomegalovirus serum PCR, hepatitis A, B, and C, 4th generation HIV screen), arboviral cultures (West Nile), bacterial cultures (*Rickettsia rickettsii, Francisella tularensis, Borrelia burgdorferi, Bartonella henselae* and *quintana, Coxiella burnetii, Leptospira, Treponema pallidum*), fungal cultures (*Coccidiodes, Histoplasma*), and beta‐D‐glucan assay. He did have a positive Parvovirus B19 PCR from blood (1500 IU/mL) with positive IgG, but negative IgM, and therefore felt to be a reactivation. He also had a positive Coxsackievirus AB‐B2 and AB‐B4 antibody with a titer of 1:40.

Given an unknown diagnosis, he was started on empiric antibiotics including doxycycline, meropenem, and linezolid, as well as high‐dose intravenous pulse‐dose steroids (1000 mg/24 h of methylprednisolone) for shock. Intravenous immunoglobulin (IVIG) was given for possible parvovirus or other infectious myocarditis. Testing for the Bourbon virus and Heartland virus was requested and sent to the Centers for Disease Control and Prevention (CDC) laboratory.

Over the following week, the patient was maintained on several inotropes and vasopressors, including norepinephrine, vasopressin, epinephrine, and Angiotensin II. Due to the ECMO complications of ongoing bleeding and thrombocytopenia (nadir 8000 μL) and with hemodynamic improvement, the patient was decannulated from ECMO on Hospital day 10. While supportive therapies were ongoing, he had a bronchoalveolar lavage culture that grew *Mucor* species. *Mucor circinelloides* was also detected on a microbial cell‐free DNA test from plasma (Karius, Redwood City, CA, USA). Liposomal amphotericin B and micafungin combination antifungal therapy was initiated. Computed tomography of the chest at that time showed heterogenous bilateral lower lobe consolidations consistent with pneumonia, atelectasis, and diffuse pulmonary edema. Unfortunately, he had severe complications of his high vasopressor requirement and critical illness, resulting in severe ischemia in all four extremities that would have likely led to multiple amputations.

**Figure 2:**
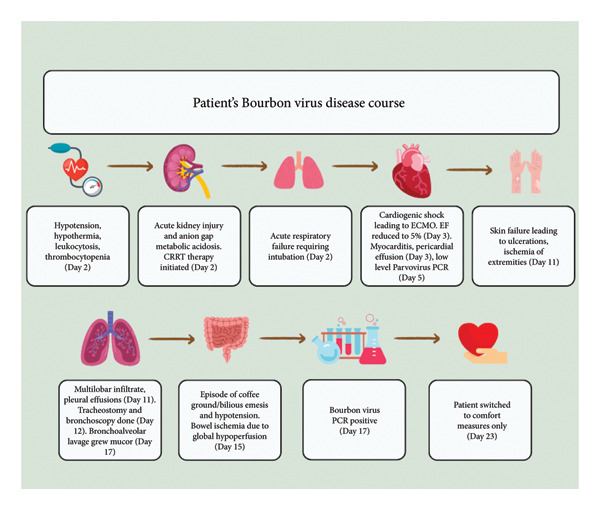
Graphical representation of the clinical manifestations of Bourbon virus in the presented patient. Day 0 refers to when the patient’s symptoms first started. Day 2 is when the patient went to urgent care.

CDC testing returned with positive Bourbon virus PCR and negative Heartland virus PCR. Due to a lack of meaningful progression and in consultation with his family, the decision was made to transition to comfort measures on Hospital day 21 (Figure 2). The patient was removed from life support and passed away peacefully. No autopsy was performed. His family provided informed consent for this case report to be published.

## 3. Discussion

**Figure 3:**
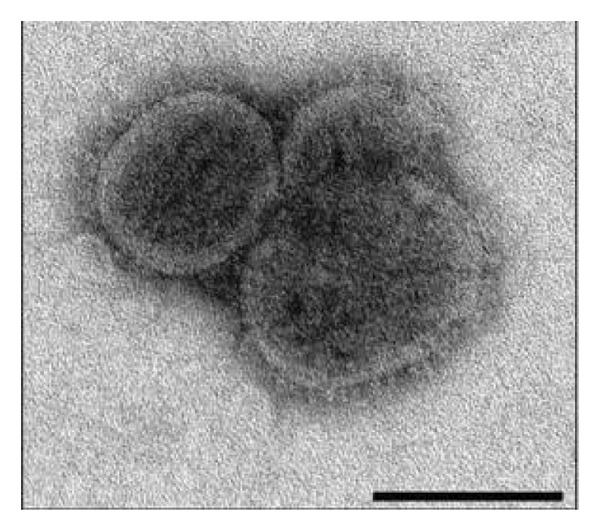
Electron micrograph of the Bourbon virus, a negative‐stranded RNA virus classified within the *Thogotovirus* genus known to be transmitted by ticks to humans. Figure courtesy of the Public Health Image Library (https://phil.cdc.gov) [6].

Bourbon virus is a negative‐sense single‐stranded RNA virus classified within the *Thogotovirus* genus, family Orthomyxoviridae (Figure 3). The genus *Thogotovirus* contains several other emerging viruses: Araguari, Aransas Bay, Dhori (including the subtype Batken), Jos, Oz, Thogoto, and Upolu viruses. Thogoto, Dhori, Oz, and Bourbon viruses are known to cause infections in humans [3, 6]. To date, the Bourbon virus is only known to be transmitted by ticks. Notably, most of the reports of symptomatic human infections associated with viruses in the genus *Thogotovirus* have had neurologic findings (e.g., meningitis and encephalitis) similar to the patient in the current report [7].

The first case of Bourbon virus involved a previously healthy male who developed symptoms several days after removing an engorged tick [7]. The patient had fever, myalgia, diarrhea, eventual multiorgan failure, and died 11 days after symptom onset from cardiopulmonary arrest. Unlike the patient presented in the current case report, this patient had leukopenia. Notably, all the patients in previous case reports of Bourbon virus presented with leukopenia, which, up until the current case report, was considered a defining sign of Bourbon virus. With the limitation of a handful of case reports, the authors postulate that it is possible that patients infected with Bourbon virus may initially have leukopenia, but over the course of their disease, later develop leukocytosis. Leukocytosis in this case could be due to evolving complications from Bourbon virus disease, specifically profound shock with elevated lactate. It is plausible that the leukocytosis represented underlying ischemia. Finally, another difference in the current case and previous reports was the development of hypothermia in our patient versus hyperthermia in previous reports [7], which could also be secondary to advanced shock, renal failure, and end‐organ damage seen in the present case. It is important to note that the patient’s hypotension, severe lower extremity edema, multiorgan failure, hemoconcentration, and shock could also be due to capillary leak syndrome (CLS). The patient did have increased inflammation with an elevated C‐reactive protein, possibly leading to increased vascular permeability and CLS [8].

In all reported cases in humans, symptoms appear around 2–7 days after tick exposure. Symptoms include weakness, nausea, myalgia, arthralgia, and diarrhea [1]. The authors of previous case reports have cited the appearance of a papular rash, although this was not present in the current case report. Previous case reports also cite elevated AST and ALT, thrombocytopenia, lymphopenia, and leukopenia. Our patient had elevated liver enzymes, lymphopenia, and thrombocytopenia. Similar to our case, all case reports describe eventual shock and cardiac dysregulation. In confirmed fatal cases, the time from initial symptoms to death was 11–24 days [1]. Another notable difference in this case is the development of pulmonary mucormycosis, likely related to steroid use in a patient with underlying diabetes and critical illness, similar to complications reported in patients with SARS‐CoV‐2 infections [9].

Over the past decade, the CDC has performed epidemiologic studies on ticks near Bourbon County, KS. The infection rate of lone star ticks with Bourbon virus infection was estimated to be 0.25–0.32 per 1000 adult ticks in 2013 and 2015 [10]. However, surveillance of ticks in 2016 failed to identify any infection of the Bourbon virus in the tested ticks. Following our patient’s diagnosis, the Kansas Department of Health and Environment surveyed the patient’s land in 2024. In total, 1253 ticks were isolated, all of which were lone star ticks, *Amblyomma americanum*. There were 207 adult and 1046 nymphal ticks isolated. These were divided into pools of 69 ticks each. One pool tested positive for the Bourbon virus, while 11 pools tested positive for the Heartland virus.

Finally, the actual diagnosis of Bourbon virus infection in humans may be underrepresented. A study analyzing the serum of 440 people near St. Louis, Missouri, found a 0.7% seroprevalence of the Bourbon virus–neutralizing antibodies in individuals, suggesting previous infection without known infection of the Bourbon virus [11]. In fact, the seroprevalence in this cohort may underestimate the true infection rate and burden since these individuals were from the greater St. Louis area, which is urban and less likely to be exposed to Bourbon virus–infected ticks. Second, the presence of detectable levels of neutralizing antibodies is a relatively high threshold for determining prior infection with any virus, including Bourbon virus, since many individuals previously infected with other viruses do not carry detectable serum‐neutralizing antibodies for long periods of time.

There is currently no known specific treatment or vaccine for the Bourbon virus because so little is known about the Bourbon virus pathophysiology. However, mice studies indicate that early antiviral treatment may be effective, including interferon‐alpha, ribavirin, favipiravir, or myricetin [1, 12, 13].

## 4. Conclusion

Bourbon virus is a tick‐borne, negative‐sense single‐stranded RNA virus that causes acute and occasionally fatal multiorgan failure in the identified cases in humans. Thus far, only six cases have been reported of Bourbon virus infection. However, due to a lack of widespread knowledge and testing, there may be other cases that have been misidentified or cases wherein symptoms are not severe enough to prompt medical attention. The patient discussed in this report presented with leukocytosis, hypothermia, cardiomyopathy, acute renal failure, rhabdomyolysis, and progressive multisystem organ failure. Initiation of VA‐ECMO allowed for ongoing therapies, but the patient still succumbed to Bourbon virus and complicating pulmonary mucormycosis. This case highlights the challenges of managing severe, novel tick‐borne illnesses. It also underscores the need for further research into novel pathogens such as the Bourbon virus, including surveillance of the only known Bourbon virus vector, the lone star tick.

## Consent

The patient’s family provided consent for this case report to be published.

## Conflicts of Interest

The authors declare no conflicts of interest.

## Funding

The authors did not receive any funding for this case report.

## References

[1] Roe M. K. , Huffman E. R. , Batista Y. S. et al., Comprehensive Review of Emergence and Virology of Tickborne Bourbon Virus in the United States, Emerging Infectious Diseases. (January 2023) 29, no. 1, 1–7, 10.3201/eid2901.212295. PMC9796205 36573641

[2] Dupuis A. P. , Prusinski M. A. , O’Connor C. et al., Bourbon Virus Transmission, New York, USA, Emerging Infectious Diseases. (January 2023) 29, no. 1, 145–148, 10.3201/eid2901.220283. 36573733 PMC9796220

[3] Ejiri H. , Lim C. K. , Isawa H. et al., Characterization of a Novel Thogotovirus Isolated From *Amblyomma testudinarium* Ticks in Ehime, Japan: A Significant Phylogenetic Relationship to Bourbon Virus, Virus Research. (April 2018) 249, 57–65, 10.1016/j.virusres.2018.03.004, 2-s2.0-85044153609. 29548745

[4] Bishop L. , ID# 28369 [Internet], 2023, https://phil.cdc.gov/Details.aspx?pid=28369.

[5] Center for Disease Control and Prevention , Public Health Imaging Library, 2023, https://phil.cdc.gov/Details.aspx?pid=28365.

[6] National Institute of Infectious Diseases and Tuberculosis and Infectious Diseases , Oz Virus Infection in Human, Infectious Agents Surveillance Report. (July 2023) 44, no. 7, 109–111.

[7] Kosoy O. I. , Lambert A. J. , Hawkinson D. J. et al., Novel Thogotovirus Associated With Febrile Illness and Death, United States, 2014, Emerging Infectious Diseases. (May 2015) 21, no. 5, 760–764, 10.3201/eid2105.150150, 2-s2.0-84928010172. 25899080 PMC4412252

[8] Siddall E. , Khatri M. , and Radhakrishnan J. , Capillary Leak Syndrome: Etiologies, Pathophysiology, and Management, Kidney International. (July 2017) 92, no. 1, 37–46, 10.1016/j.kint.2016.11.029, 2-s2.0-85015402702. 28318633

[9] Hoenigl M. , Seidel D. , Carvalho A. et al., The Emergence of COVID-19 Associated Mucormycosis: A Review of Cases From 18 Countries, The Lancet Microbe. (July 2022) 3, no. 7, e543–e552, 10.1016/s2666-5247(21)00237-8. 35098179 PMC8789240

[10] Hao S. , Ning K. , Küz ÇA. , McFarlin S. , Cheng F. , and Qiu J. , Eight Years’ Advances on Bourbon Virus, a Tick-Born Thogotovirus of the Orthomyxovirus Family, Zoonoses. (January 2022) 2, no. 1, 10.15212/zoonoses-2022-0012. PMC9206863 35727718

[11] Bamunuarachchi G. , Harastani H. , Rothlauf P. W. et al., Detection of Bourbon Virus-Specific Serum Neutralizing Antibodies in Human Serum in Missouri, USA, mSphere. (June 2022) 7, no. 3, 10.1128/msphere.00164-22. PMC9241549 35607948

[12] Hao S. , Ning K. , Wang X. et al., Establishment of a Replicon Reporter of the Emerging Tick-Borne Bourbon Virus and Use It for Evaluation of Antivirals, Frontiers in Microbiology. (September 2020) 11, 10.3389/fmicb.2020.572631. PMC7506111 33013808

[13] Bricker T. L. , Shafiuddin M. , Gounder A. P. et al., Therapeutic Efficacy of Favipiravir Against Bourbon Virus in Mice, PLoS Pathogens. (June 2019) 15, no. 6, 10.1371/journal.ppat.1007790, 2-s2.0-85068141330. PMC6564012 31194854

